# An organotypic slice model for ex vivo study of neural, immune, and microbial interactions of mouse intestine

**DOI:** 10.1152/ajpgi.00299.2015

**Published:** 2015-12-17

**Authors:** Luke A. Schwerdtfeger, Elizabeth P. Ryan, Stuart A. Tobet

**Affiliations:** ^1^Department of Biomedical Sciences, Colorado State University, Fort Collins, Colorado;; ^2^School of Biomedical Engineering, Colorado State University, Fort Collins, Colorado; and; ^3^Department of Environmental & Radiological Health Sciences, Colorado State University, Fort Collins, Colorado

**Keywords:** gut, neuroimmune, contraction, microbiome, enteric

## Abstract

Organotypic tissue slices provide seminatural, three-dimensional microenvironments for use in ex vivo study of specific organs and have advanced investigative capabilities compared with isolated cell cultures. Several characteristics of the gastrointestinal tract have made in vitro models for studying the intestine challenging, such as maintaining the intricate structure of microvilli, the intrinsic enteric nervous system, Peyer's patches, the microbiome, and the active contraction of gut muscles. In the present study, an organotypic intestinal slice model was developed that allows for functional investigation across regions of the intestine. Intestinal tissue slices were maintained ex vivo for several days in a physiologically relevant environment that preserved normal enterocyte structure, intact and proliferating crypt cells, submucosal organization, and muscle wall composure. Cell death was measured by a membrane-impermeable DNA binding indicator, ethidium homodimer, and less than 5% of cells were labeled in all regions of the villi and crypt epithelia at 24 h ex vivo. This tissue slice model demonstrated intact myenteric and submucosal neuronal plexuses and functional interstitial cells of Cajal to the extent that nonstimulated, segmental contractions occurred for up to 48 h ex vivo. To detect changes in physiological responses, slices were also assessed for segmental contractions in the presence and absence of antibiotic treatment, which resulted in slices with lesser or greater amounts of commensal bacteria, respectively. Segmental contractions were significantly greater in slices without antibiotics and increased native microbiota. This model renders mechanisms of neuroimmune-microbiome interactions in a complex gut environment available to direct observation and controlled perturbation.

the wall of the intestine is made up of five principal anatomical components with integrated and complex functional attributes. The muscle layer, known as the muscularis externa, is composed of longitudinal and circular muscle fibers, a submucosal layer, the mucosa, the gut-associated lymphoid tissue, and the enteric nervous system (ENS). The ENS is of crucial importance to translational research, principally because of its interactions across the four other components, but also because of the abundance of neurons, with roughly the same number of neurons as found in the spinal cord (14). The ENS is composed of two primary neuronal plexuses, the myenteric and the submucosal, with the myenteric plexus playing a role in peristaltic and segmental contractility, driven by the interstitial cells of Cajal (31, 33). To date, it has been difficult to capture all of these components together in functional ex vivo model systems.

Existing gut models that have been described beyond traditional in vitro intestinal cells include precision-cut intestinal slices (7, 18), gut-on-a-chip approaches (24), and microfluidic designs (35). Although there is utility in these approaches, some (7, 17) have not maintained structure beyond 24 h ex vivo or delineated a spectrum of cell types covering neural and immune components. Other studies (24, 35) did not take into account the integrated enteric nervous system, interstitial cells of Cajal, immune system functions, or the microbiome. This model incorporates the above components as well as the gut immune centers, preserved in the form of Peyer's patches, and including follicle-associated epithelia, subepithelial domes, and germinal centers. The neurons in this system are of major importance because of their ability to modulate peristaltic contractions, communicate with the brain via vagal and sympathetic pathways, and, in concert with immune cells, relay chemical signaling from bacteria of the gut (11). Although gut contractions have been described functionally and pharmacologically for many years (reviewed in Ref. 15), the lack of appropriate models has made it difficult if not impossible to tease apart chemical communications among the intestinal participants with cellular resolution. The present study adopts procedures that have been successful in brain (34), pituitary (29), and ovary (12). The present model in mice preserves the muscular, submucosal, and mucosal (crypt and villi) layers of the intestines, as well as the myenteric, submucosal plexuses, and the interstitial cells of Cajal. In addition, the structural connectivity of these components is maintained such that tissues continue spontaneous segmented contractions (as defined in Ref. 21) for up to 48 h ex vivo. Mediation of one neuronal component of these contractions and how bacteria impact them has been examined (26). However, the functional impact of the microbiome on segmental contractions has been difficult to tease apart with prior models. The present study provides an initial test of a functional impact of the presence or absence of microbiome components mediated by antibiotic usage. This functional characterization of an intestinal tissue model ex vivo provides an essential guide that should have utility for drug screening, etiology of gut disorders, teasing apart neuroimmune interactions, studying the molecular bases for enteric pathogenicity of select agents, and a foundation for gut investigations that include the microbiome.

## MATERIALS AND METHODS

### 

#### Animals.

Male and female adult mice (∼8–12 wk old) of the C57BL/6 background were used. Mice were housed in the Painter Center building under the care of Laboratory Animal Resources at Colorado State University. Mice were kept in plastic cages with aspen bedding (autoclaved Sani-chips; Harlan Teklad, Madison, WI) under a 14:10-h light-dark cycle with regular access to food (no. 8640; Harlan Teklad) and water. Intestinal slices were initially created using several transgenic strains (4, 13); however, a majority of the experiments were focused on animals expressing yellow fluorescent protein (YFP) driven by a Thy-1 promoter construct that has been suggested to be neuronal selective (10). Other Thy-1 promoter constructs lead to expression in other tissues (e.g., thymus cell antigen; 37). Animal usage was approved under Colorado State University IACUC protocol 14-5128a.

#### Organotypic slice preparation.

Adult mice were deeply anesthetized with isoflurane and killed by cervical dislocation. The small intestine was removed from the pylorus-duodenal junction to the distal ileum, and the colon, excluding the cecum, was removed. Tissue was placed immediately in 4°C 1× Krebs buffer (in mM: 126 NaCl, 2.5 KCl, 2.5 CaCl_2_, 1.2 NaH_2_PO_4_, 1.2 MgCl_2_), and dissected free from external vasculature and remaining mesenteric attachments. Successful cutting was notably dependent on minimizing mesenteric remnants prior to embedding in agarose. Slices were prepared from 1- to 3-mm sections of jejunum, ileum, and colon that were cut from the whole intestine and submerged in 8% agarose (type VII-A; Sigma; 39°C). The tissue spent a total of 7 min in the agarose: 5 min on a room temperature shaker, and 2 min in 4°C to ensure polymerization. Agarose encapsulated the entire tissue but did not penetrate the luminal space. Once the agarose was hardened, the tissue was cut at a thickness of 250 μm on a vibrating microtome (VT1000S; Leica Microsystems, Wetzlar, Germany). Slices were collected in 4°C Krebs buffer and transferred to 5 ml of Hibernate media (Life Technologies, Grand Island, NY) with 1% penicillin-streptomycin (PS; HyClone Laboratories, Logan, UT) in a 60-mm plastic-bottom dish (Corning, Corning, NY) and left at 4°C for at least 15 min. After Hibernate media, samples were transferred into 5 ml of Adult Neurobasal media (ANB; Life Technologies) with 1.3% PS and 5% B-27 supplement (Life Technologies; https://www.thermofisher.com/order/catalog/product/17504044) and incubated at 37°C for 35 min. Once initial media treatments were completed, the samples were plated on 35-mm-diameter plastic- (Corning) or glass (MatTek, Ashland, MA)-bottom dishes, with excess media being siphoned from the dish surface. Tissue was left at 37°C to adhere to the dish surface for 10 min before being covered by a thin layer of collagen [vol/vol: 10.4% 10× MEM, 1.9% PS, 4.2% sodium bicarbonate, and 83.5% collagen (PureCol; Inamed, Fremond, CA)]. Finally, the tissue was incubated at 37°C for 20 min to allow the collagen solution to polymerize before a final addition of 1 ml of ANB with PS and B-27 prior to being left in 37°C in a 5% CO_2_ incubator until visualization or experimentation. Fresh media changes were performed every 2 days.

#### Live slice imaging.

Samples were imaged at 0, 24, 48, 72, 96, 124, and 148 h ex vivo on a Nikon Te2000-U inverted microscope (×4 and ×10 Plan-Fluor objectives) with a Quantix 57 frame-shift camera (Photometrics, Tucson, AZ) and UniBlitz shutter system (Vincent Associates, Rochester, NY). Time-lapse video microscopy was used for samples that were contracting, with images collected at 500-ms intervals. A single contraction count was measured as an intestinal contraction and subsequent relaxation to equal one count. Contractions per minute were recorded and analyzed with Metamorph Microscopy Automation and Image Analysis Software (Molecular Devices, Sunnyvale, CA).

#### Nicardipine.

Calcium ion channel blocker, nicardipine (Sigma-Aldrich, St. Louis, MO) was used to clarify the origins of the slice contractions. Nicardipine was diluted in distilled H_2_O from an initial concentration of 10 mM for use at 1, 3, and 10 μM. Contractions per minute were measured by time-lapse video microscopy, with all contraction counting performed by a researcher blinded to treatment condition. Video images were collected before drug treatment and again 30 min after drug treatment. Only slices that showed contractions were used for drug treatment. Distilled H_2_O vehicle (10 μl) was used as a control. In addition, samples were washed for 1 h, four times in ANB+B-27+PS, and allowed to sit for an additional 2 h after initial washes, prior to a second drug addition. Dishes were varied for the second drug addition compared with initial treatments and were again allowed to incubate for 30 min after treatment prior to imaging. Data were collected from all dishes, including those given a second drug addition postwashing, and contraction rates were remeasured postwashing to ensure the tissue had recovered to predrug contraction rates prior to the second drug addition.

#### Cell death.

Cell death was estimated by using the membrane-impermeable red fluorescent DNA marker ethidium homodimer (EtHD; Biotium, Hayward, CA). EtHD was added to the media at a concentration of 2.5 μM, achieved with a volume of 1 μl of EtHD per 1 ml of media (ANB+PS+B-27) for 45 min, and was then washed out. Slices were then imaged on the Nikon Te2000-U inverted microscope setup at 0-, 24-, 48-, and 72-h intervals. Analysis of cell death was performed with ImageJ Image Processing and Analysis software (NIH) to determine the area of EtHD fluorescence within defined regions of interest (ROIs). Three regions were defined based on the anatomy of apical and basilar villi and the adjacent crypt regions. These regions were analyzed independently via the “analyze particles” tool on a threshold image.

#### Cell proliferation.

The incorporation of 5-ethynyl-2′-deoxyuridine (EdU; Invitrogen, Eugene, OR) was used to indicate the synthesis of new DNA in presumptive dividing cells ex vivo. Mice were injected (25 mg/kg ip) 24 h prior to euthanasia and used for intestinal slice visualization between 0 and 24 h ex vivo. Some slices were exposed to 5 μl of EdU per 1 ml of media, in vitro. Slices were then incubated for 24 or 48 h before being visualized. The EdU visualization procedure began with three phosphate-buffered saline (PBS) washes for a total of 30 min. Next, samples were placed in glycine (Fisher Scientific, Pittsburgh, PA) for 30 min before being again washed in PBS (in mM: 42.98 Na_2_HPO_4_, 7.25 NaH_2_PO_4_, 145.45 NaCl) for 10 min (1 change). Samples were then blocked with 3% bovine serum albumin buffer (BSA; Lampire Biological, Pipersville, PA) and 0.5% Triton-X (Tx) for 2 h. Samples were subsequently washed two times with 3% BSA buffer. Next, click-IT cocktail (1× click-IT Reaction Buffer, CuSO_4_, Alexa-Fluor azide, 1× reaction buffer additive; Invitrogen) was added for 2 h. Finally, slices were washed in 3% BSA buffer and 0.02% Tx three times for a total of 1.5 h and left overnight in 3% BSA before being mounted, coverslipped with Aqua-Poly/Mount (Polysciences, Warrington, PA), and imaged by confocal microscopy (Zeiss Meta 510; Carl Zeiss). Quantification of EdU incorporation was done in ImageJ (NIH) as noted for EtHD. Individual villi and crypt regions were selected from slices that demonstrated normal ileal villi structure (tall and fingerlike), with three ROIs being quantified: apical villi, basilar villi, and crypt regions.

#### Whole-mount immunohistochemistry.

Following live viewing and image collection, slices were immersion fixed in 4% paraformaldehyde for 15 min, and washed in 0.05 M PBS (pH 7.5), prior to immunohistochemical studies. Tissue processing was similar to that previously described (25). Once fixing and PBS washes were complete, the slices were incubated at 4°C in 1% sodium borohydride for 2 h. Slices were then washed in PBS for 10 min prior to incubation in block containing PBS with 5% normal goat serum (NGS; Lampire Biological, Pipersville, PA), 3% hydrogen peroxide and 0.3% Tx for 2 h with a change of solution at 1 h. Slices were placed into primary antisera; anti-NeuN (a neuronal nuclear marker) for neuronal phenotyping (Cell Signaling Technologies, Danvers, MA), neuronal nitric oxide synthase (nNOS; ImmunoStar, Hudson, WI), CD3 and CD79a antibodies (to cell surface markers for T and B cells, respectively; Novus Biologicals, Littleton, CO), and anti-c-Kit (ACK2; Novus Biologicals), with PBS containing 5% NGS and 0.3% Tx for 6 days. ACK2 was added at 2 μg/ml to live slices 90 min prior to fixation and then processed as noted for the other antisera below. NeuN primary antibody was used at 1:1,000 (bright field) and 1:200 (fluorescence) concentrations, as well as a blank for control, with all other antibodies being done at either 1:300 (CD3 and CD79a) or 1:10,000 (nNOS) concentrations. Six days following primary antibody addition, slices were washed at 4°C in PBS with 1% NGS and 0.2% Tx four times at 30-min intervals. Slices were incubated for 24 h in a biotinylated secondary antiserum (anti-rabbit, 1:2,500 or anti-rat 1:1,000 for ACK2 rat monoclonal antibody; all secondary antisera from Jackson ImmunoResearch, West Grove, PA) specific to the species of the primary antibodies and were constituted in PBS with 1% NGS and 0.32% Tx. Slices were washed for 2 h in room temperature PBS with 0.02% Tx four times before being placed in either their tertiary conjugated antibody (Cy-3) for 3 h before being washed in PBS and mounted, or Avidin-Biotin Complex (ABC; Vector Laboratories, Burlingame, CA) with 0.32% Tx/PBS for 3 h. After the ABC incubation, slices were washed in PBS at room temperature for 2 h with four changes before being placed into 0.025% diaminobenzidine (DAB; Sigma-Aldrich) in PBS; 15 min after the addition of DAB, 1% H_2_O_2_ was added for 20 min. Finally, the slices were washed three times with PBS prior to being mounted on slides and coverslipped with an aqueous mounting medium (Aqua-Poly/Mount, Polysciences, Warrington, PA).

#### Test of microbiome contribution to segmental contraction and bacteria visualization.

In a separate experiment, slices were created as noted above except that PS was omitted randomly in ∼25% of the sections to allow for a subset of native bacteria to survive under ambient oxygen conditions. Contractility rates were measured in ileum slices that either received a 1× PS dose (0.24 mM penicillin, 0.23 mM streptomycin), a 2× PS dose (0.48 mM penicillin, 0.46 mM streptomycin), or a 4× PS dose (0.96 mM penicillin, 0.92 mM streptomycin) for 24 h or were never treated with PS and imaged 24 h ex vivo. Time-lapse video microscopy was performed to count contractions, similar as with nicardipine-treated tissue. Analysis was performed using Metamorph imaging software. At 24 h ex vivo, 97% of all slices showed contractions.

Fluorescent staining was performed by using LIVE BacLight Bacterial Gram stain (Life Technologies). Equal volumes of SYTO 9 (*component A*) and hexidium iodide (*component B*) were mixed thoroughly on the day of use to create the final staining solution, and 3 μl of the staining solution was added to 1 ml of ANB+B-27 without PS. Samples were incubated in the dark for 15 min at room temperature, with subsequent media washes occurring two times for 5 min each prior to imaging. Because of technical limitations of accurately assessing the bacterial load with captured images, the density of bacteria was evaluated by using a subjective scale, with the researcher being blinded to treatment. Subjective ratings ranged from 1 (virtually no bacteria) to 4 (extremely dense bacteria).

#### Statistics.

EdU and EtHD data were analyzed by two- or three-way ANOVA for treatment and time × region with analyzed regions along individual villi considered as a “repeated measure.” PS vs. non-PS data was analyzed by one-way ANOVA, as were ratings of bacterial load. All data are presented as means (SD) and *P* < 0.05 was considered statistically significant.

## RESULTS

**Fig. 1.**
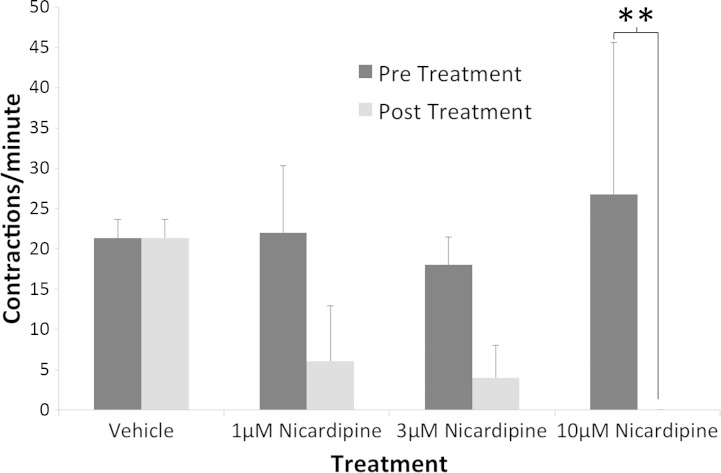
Administration of nicardipine altered the contraction rates of intestinal slices in a dose-dependent manner; 10 μM nicardipine significantly abolished contraction rates in organotypic intestinal slices compared with their predrug treatment rates (*P* < 0.001), and 1 μM and 3 μM nicardipine were intermediate in effectiveness. Distilled H_2_O vehicle (10 μl) control showed no significant change in contraction rates. **P < 0.01. Values are means (SD) (*n* = 4 slices from 4 animals).

**Fig. 2.**
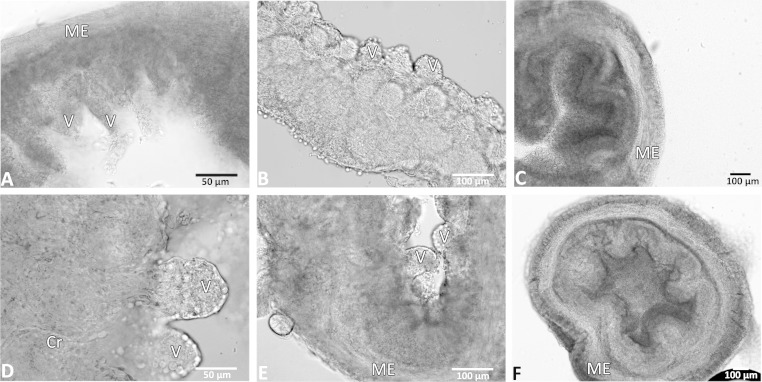
Structural integrity of organotypic slices was maintained for up to 6 days ex vivo. Bright-field images of representative organotypic slices from 3 different mouse intestinal regions at 48–144 h ex vivo, in the presence of nicardipine. Intact muscle wall, submucosa, and villi are shown, with organized enterocytes at the luminal surface. Intestinal slices at 48 h ex vivo: ileum (*A*), jejunum (*B*), and colon (*C*). *D*: higher magnification shows detailed enterocyte and crypt structures in an ileum slice 96 h ex vivo. *E*: ileum slice at 120 h ex vivo. *F*: colon slice at 144 h ex vivo. ME, muscularis externa; Cr, crypt region; V, villi. Scale bars are 50 μm in *A* and *D* and 100 μm in *B*, *C*, *E*, and *F*.

Organotypic slices maintained the structure of key components of the small intestine and colon at 250-μm tissue thickness up to 6 days ex vivo, surviving well beyond 48 h in the presence of nicardipine to block contractions (Fig. 1 and additional details below). The longitudinal and circular muscle layers were maintained with myenteric plexus frequently intact between them. In addition, the submucosal layer, including its neuronal plexus, as well as the crypts and villi of the mucosa were preserved as the epithelia showed organized enterocytes at the luminal surface (Fig. 2). Regional differences were observed, as expected, between the jejunum, ileum, and colon. Most notably, the colon lacked true villi and instead contained abundant crypt networks, compared with the long villi of the jejunum and ileum. In addition, the ileum and parts of the jejunum contained Peyer's patches, where the colon did not.

Contracting tissue was observed and contractions were counted from Metamorph time-lapse videos based on when intestines contracted and subsequently relaxed to equal one count. Slices showed an average rate of 22 (SD 4) contractions per minute in the absence of nicardipine (but with PS). In pilot experiments, intestinal tissue slices displayed continued contractions for up to 48 h ex vivo. Although the slice contractions were useful for establishing normal function relative to certain aspects of intestinal motility, the contractions negatively affected mucosal integrity after 48 h. Significant degradation, as measured by detached mucosal layers and disorganized enterocytes on the villi's luminal surface, occurred in the mucosa beyond the 48-h time point in contracting tissue. This contractility was blocked by the calcium ion channel blocker nicardipine. Initial predrug measurements, exposed to PS, were taken with an average rate of 22 (SD 4) contractions per minute. Treatment with 1 μM nicardipine resulted in a 73% decrease in contractility, whereas 3 μM nicardipine decreased it 78%, and 10 μM completely abolished contractions (Fig. 1). Contractility was restored after all drug treatments with media washes, and samples returned to predrug treatment contraction rates within 3 h after initial treatment. When contractility was blocked by nicardipine, subsequent tissue degradation was prevented and slices survived well ex vivo for up to 6 days (Fig. 2).

**Fig. 3.**
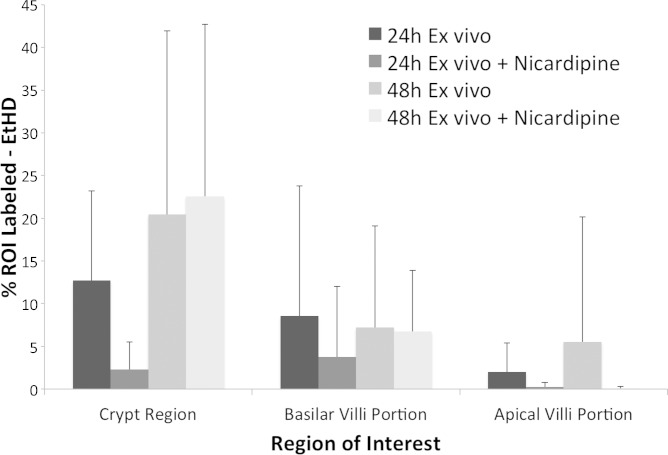
Ethidium homodimer (EtHD) labeling as indicative of dead cells varied between regions of interest (ROIs) in the villi and crypts of ileum slices. The graph shows quantification of the percentage of area labeled for EtHD between 24 and 48 h ex vivo. No significant difference in means of EtHD label was observed in specified regions of interest over the course of 48 h ex vivo (*P* > 0.1) Data are means ± SD. *N* = 9 slices from 3 animals for 24 h ex vivo, 24 h ex vivo + nicardipine, and 48 h ex vivo treatments. *N* = 15 slices from 3 animals for 48 h ex vivo treatment group.

In general, there were low levels of cell death detected in tissue slices over 2 days ex vivo (Fig. 3). EtHD and EdU data analyses in ImageJ (NIH) were based on ROI sizes that averaged 2,359 μm^2^ (SD 224; *n* = 89 slices, 6 animals). There were no significant differences in ROI sizes; repeated-measure ANOVA indicated no difference in means for EdU ROIs over the course of 0 to 24 h ex vivo (*P* > 0.60). EtHD ROIs between 24 and 48 h (*P* > 0.20) were not significantly different in mean size. The measured area labeled for EtHD suggested cell death was less than 25% in the crypt regions at both 24 h [mean = 295 μm^2^ (SD 245)] and 48 h [521 μm^2^ (SD 642)] ex vivo. Basilar villi showed less than 10% labeling with EtHD, with area labeled of 198 μm^2^ (SD 363) at 24 h and 91 μm^2^ (SD 120) at 48 h ex vivo. Apical villi demonstrated less than 5% labeling, with area labeled of 74 μm^2^ (SD 119) at 24 h and 50 μm^2^ (SD 121) at 48 h ex vivo.

**Fig. 4.**
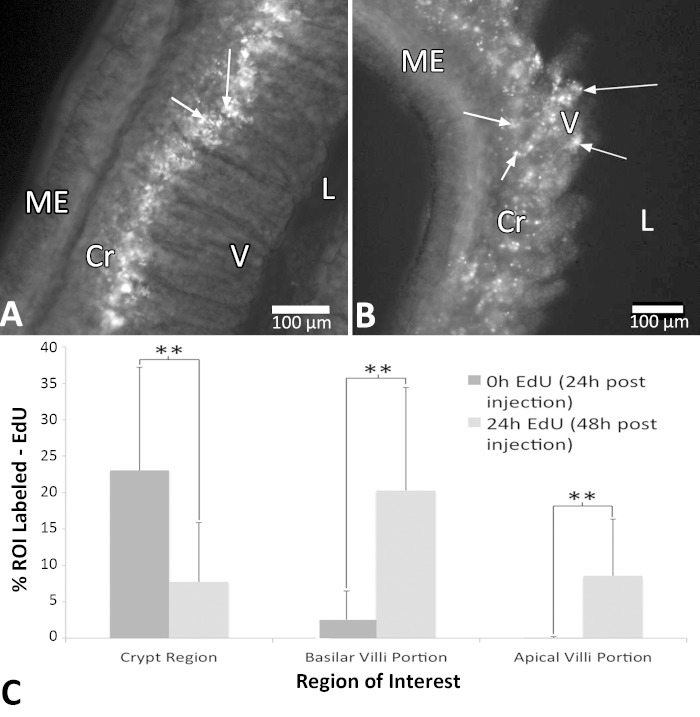
Ethinyl deoxyuridine (EdU) incorporation as indicative of DNA synthesis, proliferation, and movement varied between 0 and 24 h ex vivo. 3D montages of ileum slices are shown at 24 h (*A*) and 48 h (*B*) post-EdU intraperitoneal (ip) injection in vivo. Change in position of labeled cells is shown, with EdU uptake in the crypts and basilar portion of villi at 24 h, whereas at 48 h labeled cells appear along the entirety of the villi as well as at the luminal surface. *C*: quantification of the percentage of labeled area for EdU between 0 and 24 h ex vivo (24 to 48 h post-EdU ip injection). Data in *C* are means ± SD, *n* = 39 slices from 3 animals. **Significant difference (*P* < 0.01). ME, muscularis externa, Cr, crypt region; V, villi; L, lumen. Arrows point to cells with EdU incorporation. Scale bars in *A* and *B* are 100 μm.

**Fig. 5.**
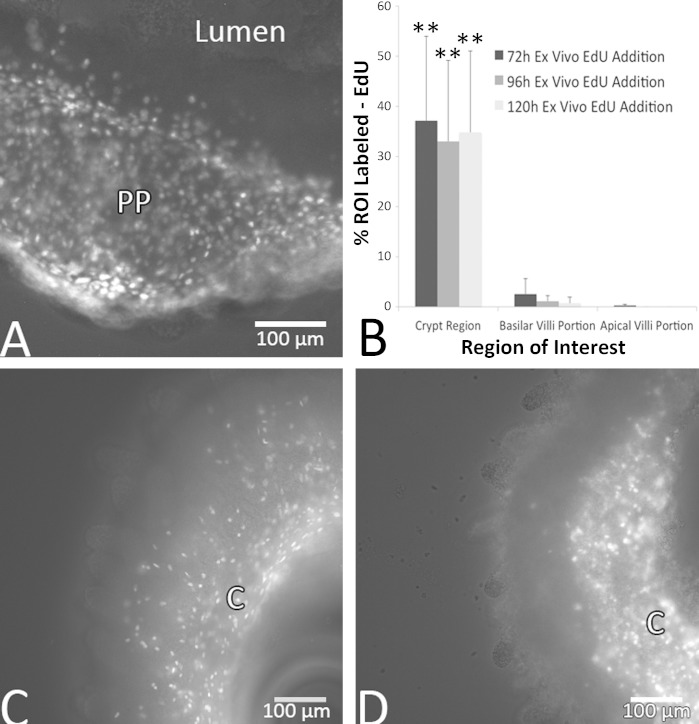
Ethinyl deoxyuridine (EdU) incorporation as indicative of DNA synthesis was observed between 96 and 144 h ex vivo after 24 h incubations with EdU. Two images taken 10 μm apart were merged by taking the maximal epifluorescent projection from ileum slices that are shown at 96 h (*A*), 120 h (*C*), and 144 h (*D*) ex vivo, 24 h after EdU addition. *A*: incorporation of EdU in a Peyer's Patch (PP). *C* and *D*: show incorporation in distal ileum slices. C, crypt regions. *B*: quantification of the percentage of labeled area of EdU in distal ileum slices at 72, 96, and 120 h ex vivo. Data in *B* are ±SD, *n* = 3 slices per time point. **Significant difference between crypt regions and both basilar and apical villi regions (*P* < 0.01). Scale bars in *A*, *C*, and *D* are 100 μm.

Evidence supporting cell proliferation and movement was provided by EdU incorporation in vivo and ex vivo (Fig. 4). In vitro EdU treatment between 24 and 120 h postslicing showed similar qualitative and quantitative labeling patterns to slices that received an ip injection 24 h prior to euthanasia (Fig. 5). When mice were injected with EdU 24 h prior to slice there was EdU incorporation and label in the crypt regions of the mucosa at 0 h ex vivo, with the area of label of 807 μm^2^ (SD 939). The basilar regions of the villi also showed incorporation [66 μm^2^ (SD 125)], albeit at a substantially diminished level compared with the crypts. The apical villi regions closest to the lumen showed virtually no incorporation of EdU, with 0.86 μm^2^ (SD 4) labeled. However, at 48 h post-EdU injection (24 h ex vivo) there was strikingly greater label observed in the basilar [909 μm^2^ (SD 706)] and apical villi [472 μm^2^ (SD 552)] regions compared with the 24 h postinjection slices and a decrease in area labeled in the crypt regions at 48 h [346 μm^2^ (SD 238)], suggesting that cells newly born in the crypt regions over the course of the first 24 h became located more apically by 48 h postinjection (Fig. 4). There was a significant difference in the areas labeled by EdU incorporation between 0 and 24 h that was dependent on the region (*P* < 0.01). The difference was based almost entirely on changes in the basal and apical villi regions between 0 and 24 h.

**Fig. 6.**
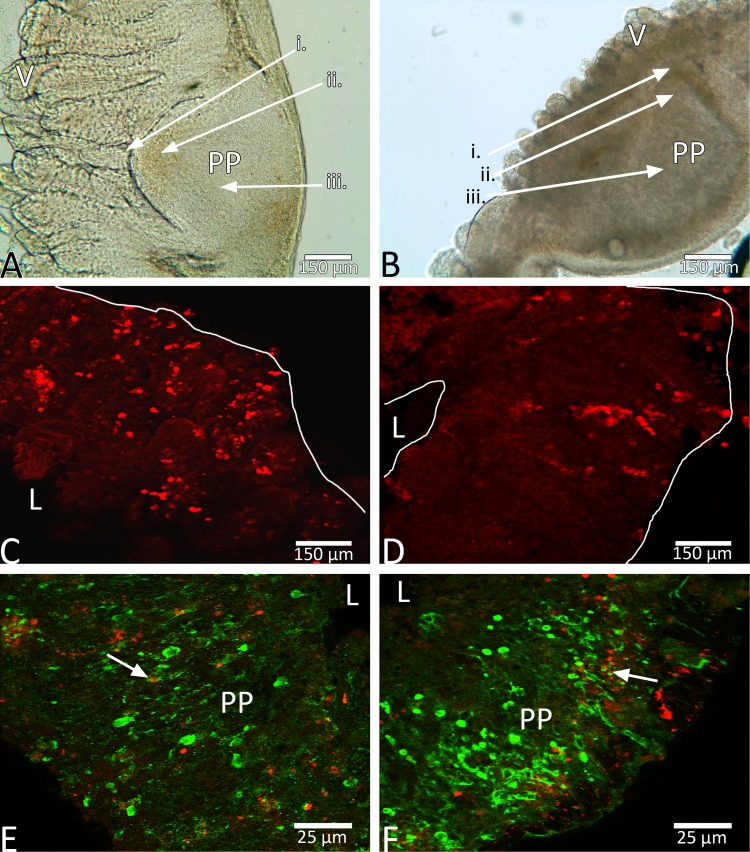
Peyer's patch integrity was maintained for 72 h ex vivo, and immunological character was demonstrated via immunoreactive CD3 and CD79a. *A*: ileum tissue 0 h ex vivo, with villi intact, muscle wall stable, and Peyer's patch maintained, including follicle-associated epithelium (FAE; *i*), subepithelial dome (SED; *ii*), and the germinal center (GC; *iii*). Example image of an ileum slice is shown at 24 h (*B*) ex vivo, again showing intact FAE (*i*), SED (*ii*), and GC (*iii*). Example fluorescent confocal images show T cells (red, CD3 in *C*) and B cells (red, CD79a in *D*) immunoreactivity in a Peyer's patch 24 h ex vivo. T cells (green, CD3 in *E*) and B cells (green, CD79a in *F*) were rarely colocalized with EtHD (red) at 72 h ex vivo. Arrows in *E* and *F* point to immunoreactive B or T cells colocalized with EtHD. PP, Peyer's patch; L, lumen. Scale bars are 150 μm in *A*–*D* and 25 μm in *E* and *F*.

Sections of intestines, primarily distal ileum, containing Peyer's patches were maintained anatomically as other non-Peyer's patch intestinal slices, with germinal centers, subepithelial domes, and follicle-associated epithelium intact (Fig. 6). Specific immunological components of these distal ileum slices containing Peyer's patches were determined via immunohistochemistry, which showed both T and B lymphocytes in abundance within Peyer's patches that were determined on the basis of gross morphology (Fig. 6, *C* and *D*). Minimal immunoreactivity for T (CD3) and B (CD79a)-cells was seen in control regions of ileum slices that were in the same slices, but not including Peyer's patches. Peyer's patch regions showed 221 (SD 182) cells labeled for CD3 and 126 (SD 68) cells for CD79a (*n* = 4 slices, two animals). By contrast, control regions situated in the intestinal wall on the opposite side from Peyer's patches had significantly less immunoreactive CD3 [58 (SD 29) cells] and CD79a [34 (SD 50) cells], demonstrating that these particular immune components of the intestinal slices were localized somewhat selectively to the Peyer's patches. Many T and B cells were visible between 24 and 72 h ex vivo based on immunohistochemistry for CD3 and CD79a (Fig. 6, *E* and *F*). When combined with EtHD for dual-label analyses of likely dying cells in Peyer's patches, there was minimal evidence of colocalization of the membrane-impermeable DNA-binding EtHD with immunoreactive T or B cells, with cells only occasionally being colocalized for both.

**Fig. 7.**
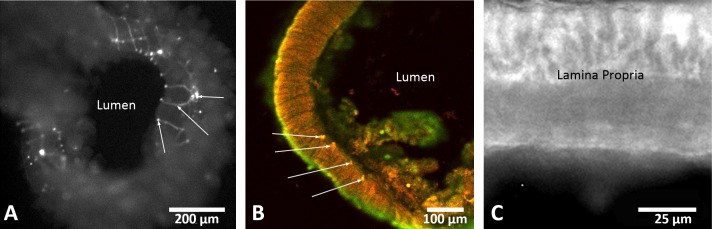
Fluorescent images from representative ileum slices show putative neuronal cells and interstitial cells of Cajal. Images in *A* and *B* use excitation of transgenic yellow fluorescent protein (YFP) driven by the Thy-1 promoter and in *B* combined with immunoreactive NeuN in the myenteric and submucosal plexuses. The dual excitation image of YFP and Cy3 (red) from NeuN immunoreactivity in *B* is taken from a slice at 24 h ex vivo. Arrows in *A* point to neuronal ganglia (*right*), axon (*middle*), and axon terminal (*left*). Arrows in *B* point to 4 different dual-labeled ganglia. *C*: immunoreactive c-Kit was localized by using the ACK2 antibody in live tissue after 48 h ex vivo. Scale bars are 200 μm in *A*, 100 μm in *B*, and 25 μm in *C*.

Several lines of evidence demonstrated intact components of the enteric nervous system in jejunum, ileum, and colon slices ex vivo. Transgenic mice in which the neuron selective Thy-1 promoter drives YFP expression (34) provided a view of neuronal networks in the live enteric nervous system (Fig. 7, *A* and *B*). After fixation and processing, immunoreactive NeuN and nNOS were found in the myenteric ganglion and plexus as a whole, with immunoreactive NeuN being localized to the soma of the neurons in these regions. These neuronal results were colocalized with images of the same slices and neuronal plexuses in Thy-1 YFP-containing tissue. In addition to immunoreactive NeuN and nNOS, intestinal slices showed ACK2 immunoreactivity (representing c-Kit in interstitial cells of Cajal) for up to 48 h ex vivo (Fig. 7*C*). Fluorescent myenteric and submucosal neuronal plexuses were seen in contracting and noncontracting ileum slices (Fig. 7*A*).

**Fig. 8.**
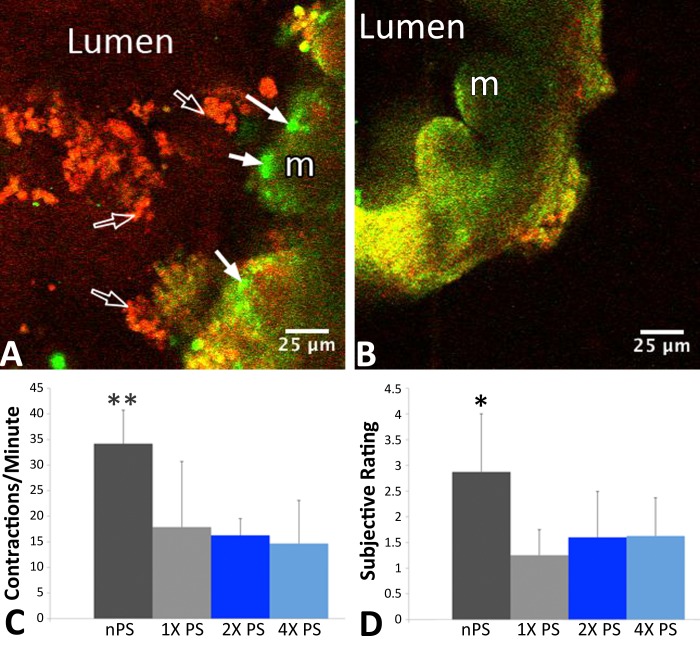
Microbial presence altered slice contractility, and penicillin-streptomycin (PS) treatment diminished bacterial density. Fluorescent Gram's stains show native bacteria in the lumen of an organotypic ileum slice. Green fluorescent SYTO 9 marked gram-negative cells (solid arrows), whereas red fluorescent hexidium iodide nucleic acid stain marked gram-positive cells (open arrows). *A*: PS-free ileum slice 24 h ex vivo showing gram-negative bacteria (green) as well as gram-positive bacteria (red). *B*: PS-treated ileum slice 24 h ex vivo showing substantially less gram-negative or gram-positive bacteria. *C*: PS-free (nPS) slices showed significantly (*P* < 0.01) greater contractions per minute than slices that saw PS for 24 h ex vivo. *D*: subjective rating system (1–4; 1 being virtually no bacteria, 4 being very dense bacteria), showed significantly more bacteria in nPS slices vs. 1×, 2×, and 4× PS-treated slices (*P* < 0.05). 1× PS dose, 0.24 mM penicillin, 0.23 mM streptomycin; 2× PS dose, 0.48 mM penicillin, 0.46 mM streptomycin; 4× PS dose, 0.96 mM penicillin, 0.92 mM streptomycin. **P < 0.01; *P < 0.05. Scale bars in *A* and *B* represent 25 μm.

To ascertain the impact native intestinal bacteria have on segmental motility, distal ileum tissue was treated with or without PS for 24 h ex vivo. Bacteria were visualized by fluorescent Gram's staining; both gram-positive and gram-negative bacteria were observed, demonstrating the ability of the intestinal slice model to maintain a subset of native bacteria under ambient oxygen conditions for 24 h ex vivo (Fig. 8, *A* and *B*). Slices not treated with PS regularly showed strong levels of gram-negative and gram-positive bacteria, whereas PS-treated slices showed significantly less bacterial load with the fluorescent Gram's stain components [Fig. 8*D*; F(3,21) = 4.3, *P* < 0.05, *n* = 9 slices for untreated (nPS), 8 for 1× PS, 4 for 2× PS, and 9 for 4× PS]. In addition to differences in commensal bacteria, slices showed significantly different segmental contractility rates. In the absence of any PS treatment (and thereby greater levels of microbiota), tissue showed a mean contraction rate of 34 (SD 7) per minute. By contrast, the mean rate for PS-treated (1× PS) slices with significantly reduced bacterial presence was 18 (SD 13) contractions per minute, 16 (SD 3) for 2× PS, and 15 (SD 8) for 4× treated slices. There was a significant difference among the nPS contraction rates and those of all three (1×, 2×, 4×) PS treatments, as measured by a one-way ANOVA [Fig. 8*C*; F(3,24) = 6.93, *P* < 0.01, *n* = 9 slices for nPS, 8 for 1× PS, 4 for 2× PS, and 9 for 4× PS]. Post hoc comparison between each PS group and the nPS group using a Fisher's least significant difference test indicated significance in each case (*P* < 0.01) and no significant differences among the PS groups.

## DISCUSSION

The present study characterizes a functional organotypic slice model for ex vivo study of the neural, immune, and microbiota components of the mouse small and large intestine. Maintaining structural integrity beyond 24 h has been difficult in previous models (6, 7) with studies lacking important cell type analyses and functional characterizations. The present model provides temporal viability with maintenance of structural integrity for all primary components of the intestine, in the jejunum, ileum, and colon at 250 μm for up to 6 days. In addition to structural and long-term temporal integrity, normal cellular processes were also maintained. For example, EdU incorporation in the crypt regions of the small intestine was consistent with previous models (16). Complete renewal of intestinal epithelium occurs in roughly 2–3 days in the mouse (3) mediated by Lgr5-expressing cells (2). Migration of these proliferating crypt cells' progeny is expected (1), with cells moving toward the villi (24), and was shown in slices in the present study. Crypt cells showed uptake and incorporation of EdU at 0 h (24 h after injection of EdU), with label also appearing in cells of the basilar portion of the villus, whereas minimal label appeared in the apical villi regions. Tissue treated with EdU at 24 h ex vivo (48 h postinjection) showed cellular fluorescence in the crypts and incorporation of EdU in cells along the entirety of the villus, including the apical villi surface. These results demonstrate the ability of the newly born crypt cells to migrate out into the apical villi regions within 48 h, which is consistent with expectations for intestinal epithelium. In addition, these EdU incorporation patterns were similar in vitro up to 120 h and in the presence of nicardipine. These results showed the capacity of the intestinal tissue slice methodology to maintain normal proliferation and migration patterns of in vivo intestinal epithelium and enterocytes in an ex vivo model system. Finally, in the context of showing maintenance of commensal bacteria with the intestinal tissue slice, a potential influence of microbiome on segmental contractions was revealed.

Peyer's patches of the ileum and their immune cellular integrity were maintained ex vivo. In the present study, intestinal slices maintained expression of both B and T lymphocytes ex vivo as shown in vivo (22). The ex vivo slices also maintained this follicle-associated epithelium, as well as the germinal center and the subepithelial dome. With Peyer's patches maintained, the present model provides for the study of intestinal immune responses in a three-dimensional, physiologically relevant environment. Intact Peyer's patches could be important for investigating responses to infections in future experiments.

YFP expression, driven by the Thy-1 promoter has previously shown its utility in neuronal migration studies in the brain (25). YFP expression in the intestines provided for visualization of myenteric and submucosal plexus putative neurons ex vivo. To confirm that the YFP-expressing cells in the intestinal slices were neuronal, immunohistochemical studies were performed for NeuN and nNOS. Large subpopulations of neurons containing nNOS have previously been shown in the myenteric and submucosal plexuses of the mouse intestine (32). These observations were confirmed in the present study, with immunoreactive nNOS being found in the myenteric ganglia and the submucosal plexus. The immunoreactive nNOS images were consistent with the YFP-expressing cells imaged live, with ganglia being localized between the circular and longitudinal muscle layers of the intestinal slices. In addition to nNOS, immunoreactive NeuN was observed in the neuronal nuclei of myenteric neurons isolated between the muscle layers of the gut and found in the ileum and the colon. NeuN has previously been shown to be a selective marker for neuronal nuclei, in the small and large intestine, and is a feature of intestinal Dogiel type II neurons (36).

Contractility ex vivo is consistent with the tissue functioning in relatively normal fashion, with contraction rates in PS-free slices within 25% of in vivo mouse peristalsis studies (39). These contraction rates, coupled with visible neuronal networks, provide elements that are advantageous in studying the myenteric plexus, the interstitial cells of Cajal, the submucosal plexus, and the mechanisms by which they receive stimuli. Unfortunately, these contractility patterns are not advantageous in maintaining general tissue structure past 48 h, with tissue showing detached or degraded mucosal layers. Calcium ion channel blockers, such as nicardipine, have been shown to block slow wave action potentials, mediated by myenteric interstitial cells of Cajal (ICC-MY) (20), and to block high-voltage-activated currents in ICC-MYs of the murine small intestine (40). In addition, dihydropyridines such as nicardipine have been shown to bind reversibly (38) to calcium channels. In the present study, nicardipine showed a dose-dependent ability to block slow wave action potentials in a reversible manner, with media washes recovering slice contractility. These results demonstrate the contractile nature of the organotypic intestinal slices to likely be a result of smooth muscle contraction, mediated by ICC-MYs. To confirm the presence of ICC-MYs in the present model system the ACK2 monoclonal antibody was used in live slices to identify them for up to 48 h in vitro. The results suggest that the ex vivo model may better maintain interstitial cells of Cajal than has previously been shown to morphologically change in vitro under other conditions (27). Furthermore, it identifies ICC-MY cells as likely mediators of the segmental contractions observed in the ex vivo model characterized in the present study as suggested by others (20, 29).

The gut microbiome has previously been shown to influence numerous cellular processes, including altering excitability of certain neuron types (26) and altering intestinal glial cell homeostasis (23). The present study addressed the hypothesis that signals from commensal bacteria impact gut function and suggests the specific ability to influence segmental motility. This agrees with recent studies using in vivo models that also demonstrated dysmotility in the presence of antibiotic treatments that impact commensal bacteria (17, 28). However, the bacterial composition of the microbiome tends toward hypoxic, with O_2_ levels in the gut being substantially lower than ambient oxygen concentrations (19). Added to that, there is a significant percentage of the microbiota that are anaerobic, with varying numbers in relation to section of the gut and location within the mucosa/luminal layers (5). Therefore the present experiment is strongly suggestive but highly preliminary in working toward a fuller picture in an ex vivo slice model of the chemical signaling that impacts motility.

In conclusion, this report presents a new model for the preparation and maintenance of intestinal slices ex vivo. Previous attempts to create viable, intact, multicellular intestinal tissue ex vivo have proven to be difficult or incomplete (18). The present model provides intestinal slices that maintain and support neuronal, muscular, and mucosal structure for up to 6 days ex vivo, marked by low levels of cell death, significant cell proliferation and migration, and maintenance of intestinal contractility, and with potential cellular immune competence. This protocol provides a modality for compelling future studies, with multiple applications relevant to complex gastrointestinal functions, including how intestinal neuronal-immune networks are structured, and the interactions in a three-dimensional, physiologically relevant environment. The intestinal model shown herein should provide sufficient time to test mechanisms for essential functional outcomes following infection (30), neuroimmune interactions (8), or metabolic transformations (9). In the absence of antibiotic in the media, aerobic components of the commensal microbiome were maintained and demonstrated a significant impact on segmental contractions. Future experiments may be able to test how the gut is affected by bacterial infections and how the natural host immune system combats these infections with modulation by drug intervention. With engineering modifications to create differential oxygen exposures, the additional components of the commensal microbiome should become available for human translational mechanistic and biochemical studies.

## DISCLOSURES

No conflicts of interest, financial or otherwise, are declared by the author(s).

## AUTHOR CONTRIBUTIONS

L.A.S. and S.A.T. conception and design of research; L.A.S. performed experiments; L.A.S. analyzed data; L.A.S. and S.A.T. interpreted results of experiments; L.A.S. prepared figures; L.A.S. drafted manuscript; L.A.S., E.P.R., and S.A.T. edited and revised manuscript; L.A.S., E.P.R., and S.A.T. approved final version of manuscript.

## Supplementary Material

Movie 1

Movie 2
